# Ammonium Chloride Ingestion Attenuates Exercise-Induced mRNA Levels in Human Muscle

**DOI:** 10.1371/journal.pone.0141317

**Published:** 2015-12-10

**Authors:** Johann Edge, Toby Mündel, Henriette Pilegaard, Emma Hawke, Murray Leikis, Nicolas Lopez-Villalobos, Rodrigo S. F. Oliveira, David J. Bishop

**Affiliations:** 1 Department of Sport and Exercise Science, University of Auckland, Auckland, New Zealand; 2 School of Sport and Exercise, Massey University, Palmerston North, New Zealand; 3 Centre of Inflammation and Metabolism (CIM), Department of Biology, University of Copenhagen, Copenhagen, Denmark; 4 Department of Renal Medicine, Wellington Hospital, Newtown, New Zealand; 5 Institute of Veterinary, Animal and Biomedical Sciences, Massey University, Palmerston North, New Zealand; 6 Institute of Sport, Exercise and Active Living (ISEAL), Victoria University, Melbourne, Australia; West Virginia University School of Medicine, UNITED STATES

## Abstract

Minimizing the decrease in intracellular pH during high-intensity exercise training promotes greater improvements in mitochondrial respiration. This raises the intriguing hypothesis that pH may affect the exercise-induced transcription of genes that regulate mitochondrial biogenesis. Eight males performed 10x2-min cycle intervals at 80% $\dot{\text{V}}{\text{O}}_{2\text{peak}}$ intensity on two occasions separated by ~2 weeks. Participants ingested either ammonium chloride (ACID) or calcium carbonate (PLA) the day before and on the day of the exercise trial in a randomized, counterbalanced order, using a crossover design. Biopsies were taken from the *vastus lateralis* muscle before and after exercise. The mRNA level of peroxisome proliferator-activated receptor co-activator 1α (PGC-1α), citrate synthase, cytochome c and FOXO1 was elevated at rest following ACID (*P*<0.05). During the PLA condition, the mRNA content of mitochondrial- and glucose-regulating proteins was elevated immediately following exercise (*P*<0.05). In the early phase (0–2 h) of post-exercise recovery during ACID, PGC-1α, citrate synthase, cytochome C, FOXO1, GLUT4, and HKII mRNA levels were not different from resting levels (*P*>0.05); the difference in PGC-1α mRNA content 2 h post-exercise between ACID and PLA was not significant (P = 0.08). Thus, metabolic acidosis abolished the early post-exercise increase of PGC-1α mRNA and the mRNA of downstream mitochondrial and glucose-regulating proteins. These findings indicate that metabolic acidosis may affect mitochondrial biogenesis, with divergent responses in resting and post-exercise skeletal muscle.

## Introduction

Repeated, transient changes in mRNA content have been reported to precede increases in mitochondrial proteins in response to exercise training in human skeletal muscle [1]. Understanding the control of this gene expression would therefore seem important to understand the mechanisms responsible for adaptations to exercise training. One factor that has been reported to affect the activity-induced transcription of metabolic genes associated with substrate utilization, such as mitochondrial uncoupling protein-3 and pyruvate dehydrogenase kinase 4 [2], is the intracellular environment (e.g., low muscle glycogen content). A yet-to-be-explored hypothesis, raised by our previous research [3], is that intracellular pH (pH_*i*_) may also affect the exercise-induced transcription of genes that regulate mitochondrial biogenesis.

There is increasing evidence that acidosis can affect molecular signalling. For example, basal insulin receptor substrate-1 (IRS-1) associated phosphatidylinositol 3-kinase (PI3-K) activity has been reported to be suppressed following a small chronic decrease in the blood pH (0.11 of a pH unit) of rats [4], with a subsequent increase in mRNAs encoding ubiquitin and protease subunits [5]. In addition, ammonium chloride (NH_4_Cl) ingestion decreases the phosphorylation of mitogen-activated protein kinase (MAPK) in rat kidneys [6]. Given that MAPK is involved in one of the main signalling pathways involved in mitochondrial biogenesis, this result provides support for the hypothesis that acidosis may affect the transcription of genes regulating mitochondrial biogenesis. This would be consistent with the observation that the addition of sodium bicarbonate (NaHCO_3_) to C2C12 myotubes promotes the up-regulation of peroxisome proliferator-activated receptor coactivator 1α (PGC-1α) and some of its downstream targets (COX-II, COX-IV and cytochrome c) [7]. However, while the effects of acidosis on kidney function and acute changes in gene expression in mammalian tissue have received much attention, little is known about the effects of manipulating pH on activity-induced gene expression in skeletal muscle.

It has previously been reported that ingesting NaHCO_3_ prior to high-intensity exercise training promotes greater improvements in endurance performance [8]. It was hypothesized that this may be due to the negative effects of a decrease in pH_*i*_ on exercise-induced changes in mitochondrial function, as measured by mitochondrial respiration [3]. It was subsequently reported that rats ingesting NaHCO_3_ before physical activity (so as to reduce the activity-induced decrease in muscle pH) had significantly greater adaptations to five weeks of exercise training [9]. This included greater mitochondrial respiration [3] compared with both a control group (no training) and a placebo group (identical exercise, but ingesting sodium chloride before each session). These results raise the intriguing possibility that pH may affect skeletal muscle gene expression associated with mitochondrial biogenesis.

The purpose of this study was to manipulate intra- and extracellular pH, via an acute acid load (NH_4_Cl ingestion), and to determine the effects on both basal mRNA levels and the exercise-induced mRNA responses of important regulators of mitochondrial biogenesis, such as PGC-1α and PGC-1β, and downstream target proteins such as citrate synthase (CS) and cytochrome c (CYT-C). We also investigated the effects of an acute acid load on basal mRNA levels and the exercise-induced mRNA responses of proteins associated with acute and chronic metabolic regulation such as glucose transporter 4 (GLUT4), hexokinase (HKII), pyruvate dehydrogenase kinase 4 (PDK4) and forkhead box protein O1 (FOXO1). We hypothesized that NH_4_Cl ingestion would exacerbate the exercise-induced acidosis and be associated with a decrease in the exercise-induced mRNA response of these important regulators of mitochondrial biogenesis and metabolism.

## Materials and Methods

### Participants

Eight moderately-trained males (age 25 ± 6 y, body mass 83.7 ± 9.0, $\dot{\text{V}}{\text{O}}_{2\text{peak}}$ 47.6 ± 7.6 mL·kg^-1^·min^-1^) volunteered to participate in this study. Each participant was involved in recreational physical activity ~3 d·wk^-1^ (walking, jogging, gym), but not training for any specific sport.

### Ethics Statement

Participants were informed of the study requirements, benefits and risks before giving written informed consent. Approval for the study procedures was granted by the Massey University Research Ethics Committee. Raw data is included in the Supplementary file—S1 Dataset.

### Experimental overview

**Fig 1:**
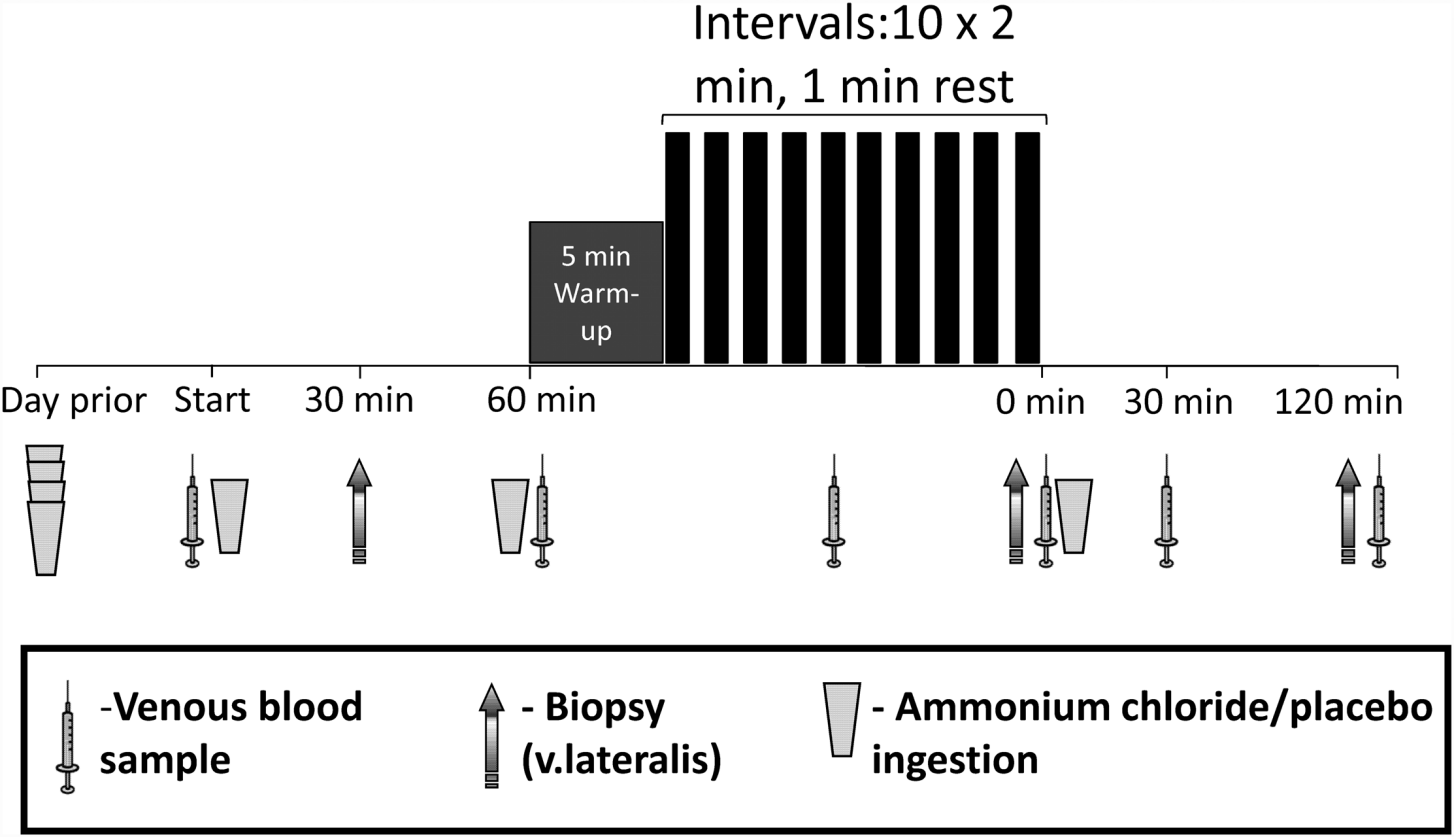
Participants ingested either ammonium chloride (0.15 g.kg^-1^, ACID) or placebo (0.15 g.kg^-1^, PLA) in four doses during the 24 h prior to the trial day. An 8 min warm-up (5 min at 40% of peak power output, 3 min at 60% of peak power output) was followed by 10 x 2 min intervals at 80% of peak power output, interspersed with 1 min of active rest at 40% of peak power output, performed on a cycle ergometer. Timings of additional supplementation, venous blood and muscle biopsy samples and a post-exercise meal are shown prior to and following the high-intensity interval session.

Participants performed the following tests: a) a maximal incremental cycling exercise test and b) two acute sessions of high-intensity interval exercise on a cycle-ergometer, combined with the ingestion of either ammonium chloride (metabolic acidosis; ACID) or calcium carbonate (placebo; PLA), in a randomized, counterbalanced order using a double-blind, cross-over design by the lead author (using sequentially-numbered envelopes). Each high-intensity interval exercise session was separated by approximately 2 weeks and all participants completed the study within < 3 weeks. A schematic representation of each acute testing day is presented in Fig 1.

### Maximal incremental cycling exercise test

The maximal incremental exercise was performed on a Lode cycle ergometer (Goningen, The Netherlands). The incremental exercise test began at 100 W, with power output increments of 25 W·min^-1^ until exhaustion [10]. Exhaustion was defined as the inability to maintain the minimum pedal cadence required (60 rev·min^-1^). In order to attain maximal values, participants received strong verbal encouragement to continue as long as possible. Expired gas was collected using Douglas Bags and measured with an Ametek analyser (Applied Electrochemistry, Pittsburgh, PA) and a dry gas meter (Harvard, UK) to determine O_2_ consumption ($\dot{\text{V}}{\text{O}}_{2}$), CO_2_ production ($\dot{\text{V}}{\text{CO}}_{2}$) and the respiratory exchange ratio (RER). The $\dot{\text{V}}{\text{O}}_{2\text{peak}}$ was considered as the highest 30-s average for $\dot{\text{V}}{\text{O}}_{2}$ obtained during the test and the peak power (PP_watt_) was determined from the last completed stage.

### Diet/exercise control

Prior to each high-intensity interval exercise session, participants were required to abstain from any vigorous physical activity for a minimum of 48 h, and were provided with a standardised meal in the 24 h prior to each trial. Meals consisted of 7 g CHO·kg^-1^ body mass, 1 g protein·kg^-1^ body mass and 2 g fat·kg^-1^ body mass. Repeated tests for the same participant were carried out at approximately the same time of day in order to control for diurnal variation [11].

### Ammonium Chloride or Calcium Carbonate ingestion

Ammonium chloride has been shown to be well tolerated at doses of up to 0.3 g.kg^-1^ [12]. In the present study, participants ingested gelatin capsules containing either ammonium chloride (ACID, 0.15 g·kg^-1^) or calcium carbonate (PLA, 0.15 g·kg^-1^), using a double blind cross-over design, the day prior to each high-intensity interval exercise session. Capsule ingestion was subdivided and occurred in four separate and equal aliquots spread throughout the day to coincide with feeding to reduce any side effects, such as gastrointestinal discomfort.

Participants then reported to the laboratory for the high-intensity interval exercise session following an ~10 h overnight fast. Additional doses of ammonium chloride and calcium carbonate (0.05 g·kg^-1^) were ingested by the participants upon their arrival in the laboratory (after a resting blood sample), 1 hour after their arrival (after a resting muscle biopsy), and immediately after the completion of the high-intensity interval exercise session. Therefore, the total ingestion of each supplement was 0.15 g·kg^-1^ on the day of the exercise protocol, equaling that consumed on the day prior to the trial. No blood or tissue samples were taken prior to the initial capsule ingestion the day before the trial.

### Exercise protocol

The high-intensity exercise session consisted of an 8 min warm-up—5 min at 40% of PP_watt_ followed by 3 min at 60% of PP_watt_. This was immediately followed by 10 x 2-min intervals at 80% PP_watt_, interspersed with 1 min of active recovery at 40% PP_watt_. Each exercise bout was completed on the same cycle ergometer as the maximal incremental test, and all participants were able to complete all intervals in both conditions.

### Blood sampling and analysis

Shortly after arriving at the laboratory, a cannula (20-G, Becton Dickinson, US) was inserted into an antecubital vein of the participants and a resting venous blood sample was taken. Further venous blood samples were taken immediately prior to, immediately after exercise, and 2 h into recovery. The venflon was kept patent with 0.9% saline (AstraZeneca, Aus). For each sample, the initial 2 mL drawn was discarded and blood was then collected into two 4.5 mL EDTA-containing tubes and one 4.5 mL lithium-heparin containing tube (Becton Dickinson, UK). Heparinised samples were analysed for pH, HCO_3_^-^ and lactate using an automated blood-gas analyser (ABL800 FLEX, Radiometer Medical ApS, Denmark). Tubes were placed on melting ice until centrifuged (2300 *g* at 4°C for 10 min) and the serum or plasma aliquotted and stored at –80°C until subsequent analysis.

### Muscle sampling and analysis

On the day of each high-intensity exercise bout, three incisions were made through the subcutaneous tissue and underlying muscle fascia under local anaesthesia (5 mL, 1% Xylocaine) into the lateral aspect of the *vastus lateralis*, about one-third of the distance from the upper margin of the patella to the anterior iliac spine of each subject. Pre and post-exercise samples were taken from one leg, while the 2 h post-exercise samples were taken from the other. *Vastus lateralis* muscle biopsy samples were taken using the Bergstrom technique with suction applied. The samples were then removed from the biopsy needle, blotted free of excess blood and rapidly placed in liquid nitrogen and stored at -80°C until subsequent analysis.

### RNA isolations, reverse transcription and PCR

Total RNA was isolated from approximately 30 mg of muscle tissue and the final RNA pellet was re-suspended in 1 μL per mg original tissue in diethyl pyrocarbonate (DEPC)-treated H_2_O containing 0.1 mM EDTA as previously described [13]. Reverse transcription (RT) was performed on 3 μg total RNA of each sample using the superscript II RNase H^-^ systemwith Oligo dT (invitrogen, Carlsbad, CA, USA) and the RT products were diluted in nuclease-free H_2_O as previously described [13]. The amount of single strand DNA (ssDNA) was determined in the RT samples using OliGreen reagent (Molecular Probes, The Netherlands) as previously described [14]. The mRNA content was determined for selected genes using fluorescence-based real time PCR (ABI PRSIM 7900 Sequence Detection System, Applied Biosystems, CA, USA). Forward (FP) and reverse (RP) primers and Taqman probes were designed from human specific sequence data (Entrez-NIH and Ensembl, Sanger Institute) using computer software (Primer Express, Applied Biosystems). The primer and probe sequences are given in Table 1. The probes were 5’ 6-carboxyfluorescein (FAM) and 3’ 6-carboxy-N, N, N’, N’-tetramethylrhodamine (TAMRA) labeled. Prior optimization was performed to determine the optimal primer and probe concentrations. PCR amplification was performed in triplicates in a total reaction volume of 10 μL with 21 ng cDNA as previously described [14]. Serial dilutions were made from a pooled representative sample and these samples were amplified together with the unknown samples and used to construct a standard curve. The obtained cycle threshold (Ct) values reflecting the initial content of the specific transcript in the samples were converted to an arbitrary amount by using the standard curve. For each sample, the amount of a given target cDNA was normalized to the ssDNA content in the sample.

**Table 1:** Primer and TAQMAN probe sequences used for real-time PCR. PDK4, pyruvate dehydrogenase kinase 4; PGC-1α, peroxisome proliferator-activated receptor-γ coactivator-1α CS, citrate synthase; Cyt *c*, cytochrome *c* oxidase, HKII, hexokinase II; GLUT4, glucose transporter-4; PGC-1β, peroxisome proliferator-activated receptor gamma coactivator-1β FOXO1, forkhead box O1.

Gene	Primer sequence (forward and reverse)	TaqMan Probe
**PDK4**	5'-TCCACTGCACCAACGCCT-3'	5'-ATAATTCCCGGAATGCTCCTTTGGCTG-3'
	5'-TGGCAAGCCGTAACCAAAA-3'	
**PGC-1α**	5’-CAAGCCAAACCAACAACTTTATCTCT-3’	5’-AGTCACCAAATGACCCCAAGGGTTCC-3’
	5’-CACACTTAAGGTGCGTTCAATAGTC-3’	
**CS**	5’-GACTACATCTGGA ACACACTCAACTCA-3'	5'-ACGGGTTGTTCCAGGCTATGGCCA-3'
	5'-CGCGGATCAGTCTTCCTTAGTAC-3'	
**Cyt *c***	5'-GGTCTCTTTGGGCGGAAGAC-3’	5'-CCCTGGATACTCTTACACAGCCGCCAA-3'
	5' CTCTCCCCAGATGATGCCTTT 3’	
**HKII**	5'-TTGTCCGTAACATTCTCATCGATT-3'	5'-ACCAAGCGTGGACTGCTCTTCCGA-3'
	5'-TGTCTTGAGCCGCTCTGAGAT-3'	
**GLUT4**	5'-CCTGCCAGAAAGAGTCTGAAGC-3'	5'-CAGAAACATCGGCCCAGCCTGTCA-3'
	5'-ATCCTTCAGCTCAGCCAGCA-3’	
**PGC-1β**	5'-GAGGGCTCCGGCACTTCT-3'	5'-CCCAGATACACTGACTACGATTCCAATTCAGAAG-3'
	5'-CATGGCTTCATACTTGCTTTTCC-3'	
**FOXO1**	5'-ACCGAACAGGATGATCTTGGA-3'	5'-CCATCTGCCGCAAAGATGGCCTCTA-3'
	5'-TTGCTTATCTCAGACAGACTGGGTAA-3'	

### pH analysis

Muscle samples were freeze dried and dissected free of blood, fat and connective tissue. The dried muscle sample (1–3 mg) was homogenized in 10 mM NaF (i.e., 1 mg: 33 μL) and then placed in a water bath at 37°C for 5 minutes. Muscle pH was determined with a microelectrode (MI-410, Microelectrodes Inc, Bedford, NH, USA) connected to a pH meter (Schott Instruments GmbH, lab 850, Mainz, Germany). The pH values were recorded at every 30 seconds but the final pH value for each sample was determined by the average of the last two minutes.

### Statistical analysis

Statistical analyses were performed using SAS (Statistical Analysis System, version 9.3; SAS Institute Inc., Cary, NC, USA). Repeated measures of the dependent variables recorded in the same subject were analyzed using the MIXED procedure fitting a mixed model that included the fixed effects of treatment (ACID AND PLA), time (rest, post-ex and 2 h), the interaction between treatment and time and the random effect of subject. Using the Akaike’s information criterion, a compound symmetric error structure was determined as the most appropriate residual covariance structure for repeated measures over time within subjects. Multiple mean comparisons of gene expressions were performed on the logarithm scale and presented in graphs after back-transformation. Significant differences between means were declared at P<0.05, and the number of participants for each analysis was n = 8.

## Results

### Blood variables

At rest, venous blood pH and HCO_3_^-^ were both lower in the ACID trial than in the PLA trial (*P* < 0.05; Table 2). Additionally, at all post-exercise time points, venous blood pH and HCO_3_^-^ were lower in the ACID trial than in PLA (*P* < 0.05; Table 2). Venous blood lactate concentration was similar at rest between trials, increased in response to exercise, but only the immediately post-exercise venous blood lactate concentration was lower in the ACID than in the PLA trial (*P* < 0.05).

**Table 2:** Least squares means (and standard errors) for plasma pH (pH), bicarbonate (HCO_3_-) and lactate (Lac^-^) concentrations for placebo (PLA) and acidosis (ACID) trials at rest (REST), immediately after exercise (POST-EX) and 2 hours (2H) following exercise.

		Rest	Post-Ex	2h
**pH**	PLA	7.385 (0.023)	7.302 (0.021)*	7.396 (0.022)‡
	ACID	7.293 (0.022)†	7.184 (0.019)* †	7.303 (0.021)* ‡ †
**HCO**_**3**_ **-** (mmoL·L^-1^)	PLA	27.2 (1.2)	20.3 (2.5)*	26.3 (1.4)‡
	ACID	17.4 (1.1)†	10.3 (0.)* †	14.1 (1.1)* ‡ †
**Lac** ^**-**^ (mmoL·L^-1^)	PLA	1.7 (0.2)	8.3 (1.2)*	2.1 (0.4)‡
	ACID	1.4 (0.2)	5.4 (0.7)* †	2.0 (0.2)‡

* Denotes significant difference from Rest value within the treatment (*p* < 0.05).

^‡^ Denotes significant difference from Post-Ex value within the treatment (*p* < 0.05).

^†^ Denotes significant difference to PLA value within the time (*p* < 0.05).

### Muscle pH

There were no significant differences in pre-exercise muscle pH between ACID and PLA (7.10 ± 0.04 vs 7.10 ± 0.04; *P* > 0.05). However, there was a significantly lower muscle pH immediately post exercise (6.86 ± 0.11 vs 6.93 ± 0.09; *P* < 0.05), and 2 h post the exercise bout (7.05 ± 0.05 vs 7.12 ± 0.04; *P* < 0.05) in the ACID trial than in the PLA trial.

### mRNA

**Fig 2:**
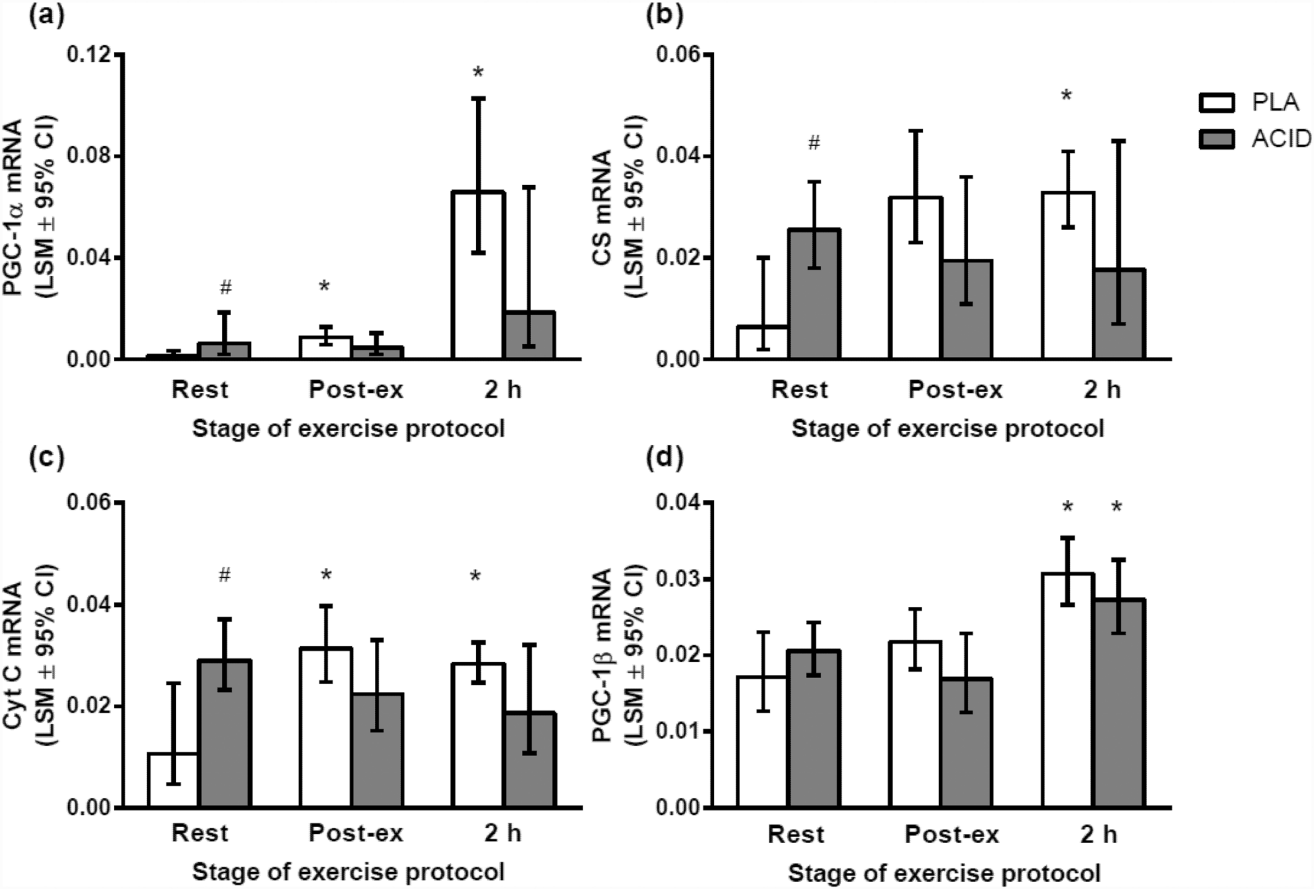
Gene expression responses to a high-intensity interval exercise bout (10 x 2 min at 80% peak power output, 1 min @ 40% of peak power output) after the ingestion of ammonium chloride (ACID) or placebo (PLA). The total ingestion of each supplement was 0.15 g·kg^-1^ on the day of the exercise protocol, equaling that consumed on the day prior to the trial. (a) PGC-1α, (b) citrate synthase, (C) cytochrome C; (d) PGC-1β. # significantly different to placebo at same time point (P < 0.05); * significantly different to same ingested substance at rest (P < 0.05). Values are least square means ± 95% confidence limits.

**Fig 3:**
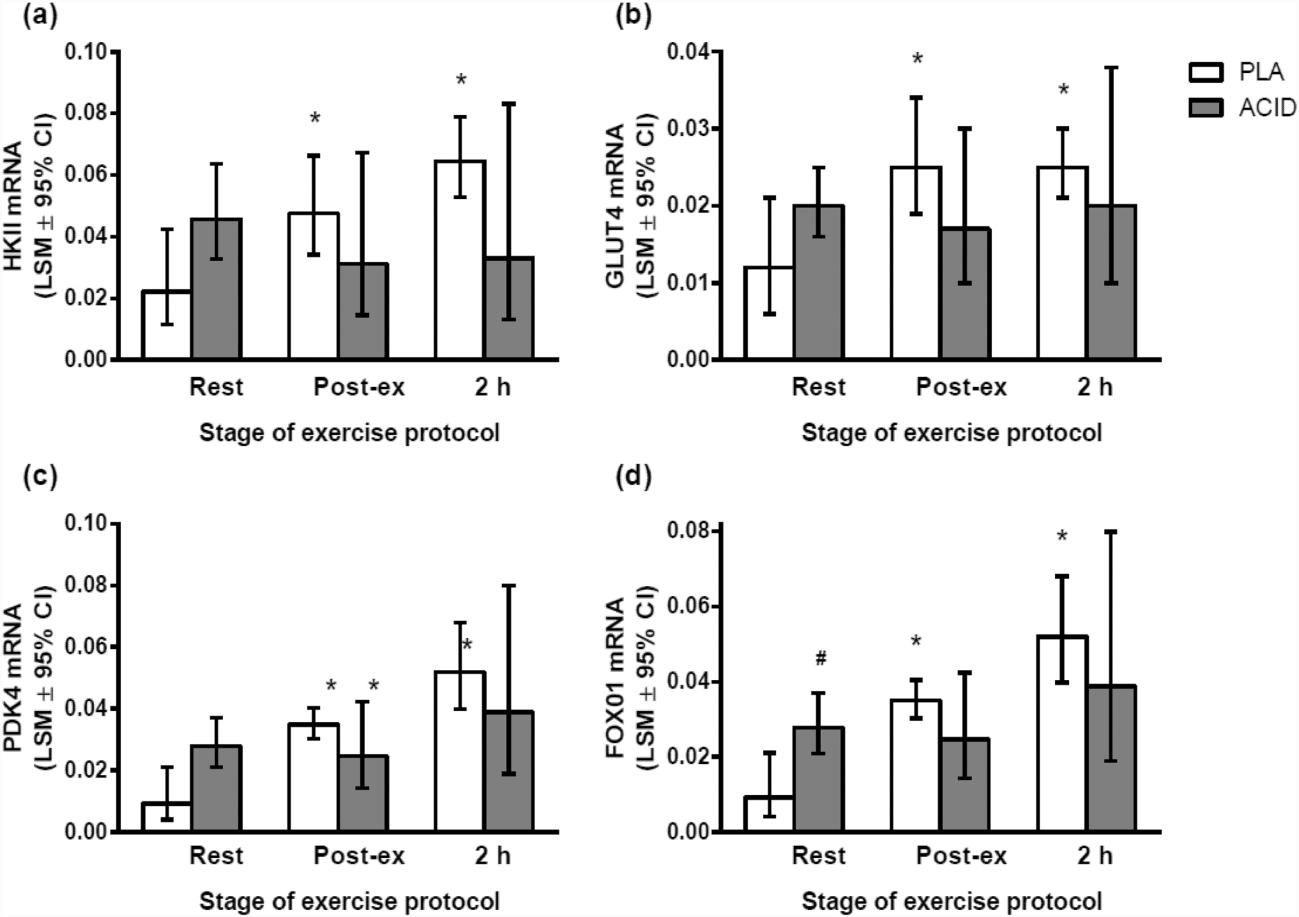
Gene expression responses to a high-intensity interval exercise bout (10 x 2 min @ 80% peak power output, 1 min @ 40% of peak power output) after the ingestion of ammonium chloride (ACID) or placebo (PLA). The total ingestion of each supplement was 0.15 g·kg^-1^ on the day of the exercise protocol, equaling that consumed on the day prior to the trial. (a) HKII, (b) GLUT4, (C) PDK4; (d) FOXO1. # significantly different to placebo at same time point (P < 0.05); * significantly different to same ingested substance at rest (P < 0.05). Values are least square means ± 95% confidence limits.

At rest there was a significantly higher mRNA content of PGC-1α, CS, CYT-C and Foxo mRNA in ACID than in PLA (*P* < 0.05; Figs 2 and 3). The resting mRNA content for HKII (*P* = 0.06) and GLUT mRNA (*P* = 0.10) was not significantly higher in ACID compared with PLA (Fig 3). There were no significant differences in PGC-1β or PDK4 at rest between the ACID and PLA trials (*P* > 0.05; Figs 2 and 3 respectively).

There was a significant increase in the mRNA content of PGC-1α, PGC-1β, CS, CYT-C, FOXO1, HKII, GLUT4 and PDK4 mRNA following the exercise bout relative to Pre during the PLA trial (*P* < 0.05; Figs 2 and 3). However, only PGC-1β was elevated early post-exercise (compared with rest) during the ACID trial (*P* < 0.05). There were no significant differences between conditions for the post-exercise mRNA values. There was also no significant difference between ACID and PLA PGC-1α mRNA 2 h post-exercise (P = 0.08).

## Discussion

The present study investigated the effects of NH_4_Cl ingestion on mRNA content at rest and following high-intensity interval exercise. For the first time, we have shown that NH_4_Cl ingestion alters basal and exercise-induced changes in the mRNA content of PGC-1α mRNA and other mitochondrial/metabolic proteins in human skeletal muscle. At rest, the mRNA content of PGC-1α and its downstream targets, such as CS and CYT-C, was greater in ACID versus PLA. However, only in the PLA condition was the post-exercise mRNA content of mitochondrial- and glucose-regulating proteins greater than resting values (*P*<0.05). In contrast, the post-exercise mRNA contents of PGC-1α, CS, CYT-C, FOXO1, GLUT4, and HKII mRNA levels were not different from resting levels in the ACID condition (*P*>0.05). Therefore, our results show that NH_4_Cl ingestion increases the mRNA content of some genes in skeletal muscle at rest, but reduces the exercise-induced response of these same genes. Hence, NH_4_Cl ingestion appears to have different effects on the resting and post-exercise mRNA content of PGC-1α and its downstream targets.

### Efficacy of ingestion protocol

Administration of NH_4_Cl has widely been used to induce extracellular acidosis [12]. NH_4_Cl uptake by the liver results in the formation of urea and the net release of HCl, which is not completely buffered by extracellular bicarbonate. As a consequence, there was a significant decrease in resting values for both venous bicarbonate and blood pH (Table 2). Despite the large decrease in resting extracellular pH, there was no change in resting muscle pH. This is consistent with previous human [12] and *in-vivo* rat studies [15], and has been suggested to be attributed to the actions of intracellular buffers (e.g., protein-bound histidine residues, imidazole-containing dipeptides and phosphates within the muscle; [16]) that act to maintain pH_*i*_. This indicates that the response of intact animals to a decrease in extracellular pH is more complex than in cultured cells where pH_*i*_ typically decreases in parallel with extracellular pH.

In contrast to the resting condition, and similar to previous results [12], the lower post-exercise extracellular pH following NH_4_Cl ingestion was associated with a significantly lower pH_*i*_. Consistent with previous research in humans [12], NH_4_Cl ingestion also resulted in significantly lower post-exercise plasma pH and blood lactate values (Table 2). The lower post-exercise blood pH can be attributed to the reduced extracellular buffer capacity, while the lower blood lactate concentration can be attributed to both inhibition of glycolysis and reduced lactate transport out of muscle cells when the extracellular pH is increased [17].

### The effects of metabolic acidosis on resting mRNA content

At rest, PGC-1α mRNA, and the mRNA content of some of its downstream targets, such as CS and CYT-C, was elevated following NH_4_Cl ingestion (Fig 2). Indeed, the resting levels of these genes were elevated to a similar degree to that observed immediately post-exercise during the PLA condition. While this is the first study to determine the effects of NH_4_Cl ingestion on the mRNA content of mitochondrial proteins in skeletal muscle, it has previously been reported that a decrease in blood pH of 0.14 pH units increases the expression of a range of genes in mice kidneys, including citrate synthase [18]. These results suggest that extracellular acidosis is associated with increased resting mitochondrial gene expression in a range of tissues.

In contrast to the effects of NH_4_Cl ingestion on mitochondrial mRNA content, there was no significant increase in the resting mRNA content of glucose-regulating genes (i.e., HKII and GLUT4; Fig 3). There was however, a significant increase in FOXO1 mRNA content (Fig 3). This is consistent with the observation that in muscle atrophying due to uremia, there is an increase in FOXO1 mRNA [19]. Furthermore, acute metabolic acidosis has been reported to increase protein breakdown in rat skeletal muscle [20], via an increase in mRNAs encoding ubiquitin and protease subunits [5,21]. Extracellular acidosis has also been reported to affect the IRS/PI3-K cascade [4]. Specifically, extracellular acidosis was associated with an increase in the amount of PI3-K p85 subunit protein which decreased IRS-1-associated PI3-K activity, resulting in a reduction in the phosphorylation (i.e., activation) of Akt (protein kinase B) [4]. As phosporylated Akt suppresses the activity of the FOXO1 class of transcription factors [22], a decrease in the phosphorylation of Akt could help to explain the increase in FOXO1 mRNA content that was observed at rest in the ACID condition in the present study. These results suggest that extracellular acidosis is a powerful stimulus up-regulating multiple pathways associated with an increase in protein degradation.

Potential mechanisms for the altered resting mRNA levels in the present study require further investigation. Nonetheless, as NH_4_Cl ingestion did not alter resting pH_*i*_, it appears likely the mechanism(s) is related to the observed decrease in extracellular pH (and/or bicarbonate). There are a number of receptors and pathways that are thought to be affected by extracellular acidosis. For example, both the system A neutral amino acid transporter (SNAT2) [23] and G-protein-coupled receptors (GPCRs) [24] are thought to be sensitive to extracellular acidosis. Activation of GPCRs may explain why p38 MAPK has been reported to be activated by a decrease in extracellular pH [25]. Relevant to our findings, p38MAPK phosphorylates PGC-1α. This increases the stability of this protein, and promotes the release of PGC-1α repressors, which improves the transcription of mitochondrial genes [26]. Intracellular free calcium concentrations also rise in whole animals and intact cells in response to ammonium chloride treatment [27]. In addition, ammonia induces an increase in reactive oxygen species (ROS) production [28]. ROS and intracellular free calcium are known messengers in the p38MAPK and Ca^2+^-calmodulin-dependent kinase (CaMK) pathways, and have also been suggested to regulate PGC-1α [29]. Further research is required to investigate the role of p38 MAPK and CaMK in responding to extracellular pH changes and increasing the expression of mitochondrial genes in human skeletal muscle.

### The effects of NH_4_Cl ingestion on post-exercise mRNA content

During the PLA trial there was a significant post-exercise increase in most of the genes that were measured (2–20 fold; Figs 2 and 3). These activity-induced increases in mRNA content are consistent with previous reports [30,31]. However, extracellular acidosis was associated with an attenuated exercise-induced expression of these genes, such that only PGC-1β was elevated 2 h post-exercise (compared with rest) during the ACID trial. Furthermore, PGC-1α mRNA content 2 h post-exercise in the ACID trial was 3-fold lower than in the PLA trial (Fig 2). These results indicate that NH_4_Cl ingestion reduces the normal post-exercise increase in mRNAs for proteins associated with mitochondrial biogenesis and glucose regulation.

It has previously been reported that the intracellular environment (e.g., low muscle glycogen) affects the activity-induced transcription of metabolic genes associated with substrate utilization, such as mitochondrial uncoupling protein-3 and pyruvate dehydrogenase kinase 4 [2,32]. We provide new information that pH may also affect the exercise-induced mRNA responses of proteins that regulate mitochondrial biogenesis (and glucose regulation). This blunting of the mRNA response may be due to the already elevated resting mRNA level of PGC-1α and other metabolic proteins and/or the effects of altered metabolite levels on signaling cascades when intense muscle contraction is accompanied by ammonium-chloride-induced acidosis.

Previous research has shown that NH_4_Cl ingestion reduces muscle AMP, ADP and lactate content following intense exercise [12,33,34]. It is well known that changes in phosphorylation potential (e.g., AMP:ATP ratio) initiate numerous downstream molecular events in skeletal muscle, at least in part via AMPK activation [35]. By sensing the energy status of the muscle cell, AMPK has been suggested to be a critical regulator involved in initiating mitochondrial biogenesis [36]. In addition, muscle lactate has also been proposed to be an important signaling molecule that affects transcription factors involved in mitochondrial biogenesis [29]. Thus, smaller exercise-induced changes in muscle AMP and lactate accumulation may contribute to our observations of smaller exercise-induced increases in mRNA content following NH_4_Cl ingestion. However, further research is required to identify the mechanisms by which NH_4_Cl ingestion blunted the normal exercised-induced increase in mRNA content in the present study.

### Exercise versus rest—why the difference?

An interesting and novel finding from the present study is that NH_4_Cl ingestion enhanced resting mRNA content, but blunted the exercise-induced increase in mRNA content. These contrasting effects may be attributable to the different effects of intracellular and extracellular pH changes. As discussed earlier, NH_4_Cl ingestion was associated with a decrease in both intracellular and extracellular pH post exercise, but a decrease in resting extracellular pH only. It may therefore be hypothesized that the increase in resting mRNA content was due to membrane components sensitive to extracellular acidosis (e.g., GPCRs; [24,37]) or the system A neutral amino acid transporter (SNAT2) [23], whereas the mechanisms explaining the blunted post-exercise mRNA response are related to changes in pH_*i*_. It is also possible that whether gene expression is up or down regulated depends on the magnitude of the pH change. It has previously been reported that the rate of vascular endothelial growth factor (VEGF) transcription was increased in cells at a pH of 7.1, compared with 7.4, but decreased at a pH of 6.9 [38]. Further research is required to investigate both of these hypotheses.

### Limitations

While administration of NH_4_Cl is a commonly-used used model of acidosis [12,39], it is possible that our results can be attributable to a specific effect of NH_4_Cl (e.g., urea production), rather than changes in pH. However, similar decreases in protein synthesis have been observed following administration of either NH_4_Cl or a cation-exchange resin [39]; the resin induces acidosis by releasing hydrogen ions in exchange for other cations in the gastrointestinal tract, thus lowering the systemic pH. While further research, using different substances to alter pH is required, these findings suggest that the altered mRNA levels in the present study are unlikely to be specific to NH_4_Cl ingestion.

### Conclusions

The present results suggest that disturbances to acid-base homeostasis have the potential to alter both resting and post-exercise mRNA content of proteins related to mitochondrial biogenesis and glucose regulation. The changes in the mRNA content of mitochondrial-related proteins are consistent with our previous research demonstrating that increasing extracellular pH prior to training is associated with significantly greater improvements in endurance performance in humans [8] and mitochondrial respiration in rats [3]. While further research is required, these results have potential implications for populations who experience a greater decrease in pH at rest (e.g., chronic renal failure patients [39]) or during physical activity (e.g., diabetics [40]).

## Supporting Information

S1 Dataset Raw data for individual participants. (PDF)

## References

[1] Perry CG, Lally J, Holloway GP, Heigenhauser GJ, Bonen A, Spriet LL (2010) Repeated transient mRNA bursts precede increases in transcriptional and mitochondrial proteins during training in human skeletal muscle. J Physiol 588: 4795–4810. 10.1113/jphysiol.2010.199448 20921196 PMC3010147

[2] Pilegaard H, Keller C, Steensberg A, Helge JW, Pedersen BK, Saltin B, et al (2002) Influence of pre-exercise muscle glycogen content on exercise-induced transcriptional regulation of metabolic genes. J Physiol 541: 261–271. 12015434 10.1113/jphysiol.2002.016832 PMC2290316

[3] Bishop DJ, Thomas C, Moore-Morris T, Tonkonogi M, Sahlin K, Mercier J (2010) Sodium bicarbonate ingestion prior to training improves mitochondrial adaptations in rats. American Journal of Physiology Endocrinology and Metabolism 299: E225–233. 10.1152/ajpendo.00738.2009 20484007

[4] Bailey JL, Zheng B, Hu Z, Price SR, Mitch WE (2006) Chronic Kidney Disease Causes Defects in Signaling through the Insulin Receptor Substrate/Phosphatidylinositol 3-Kinase/Akt Pathway: Implications for Muscle Atrophy. J Am Soc Nephrol 17: 1388–1394. 16611720 10.1681/ASN.2004100842

[5] Bailey JL, Wang X, England B, Price S, Ding X, Mitch WE (1996) The acidosis of chronic renal failure activates muscle activates muscle proteolysis in rats by augmenting transcription of genes encoding proteins of the ATP-dependent ubiquitin-proteosome pathway. Journal of Clinical Investigation 97: 1447–1453. 8617877 10.1172/JCI118566 PMC507204

[6] Bento LM, Carvalheira JB, Menegon LF, Saad MJ, Gontijo JA (2005) Effects of NH4Cl intake on renal growth in rats: role of MAPK signalling pathway. Nephrol Dial Transplant 20: 2654–2660. 16169866 10.1093/ndt/gfi133

[7] Perez-Schindler J, Philp A, Baar K (2009) Sodium bicarbonate increases glucose uptake and mitochondrial biogenesis in C2C12 myotubes potentially via the transcriptional co-activator PGC-1a. Proceedings of the Physiological Society 14: PC44.

[8] Edge J, Bishop D, Goodman C (2006) Effects of chronic NaHCO3 ingestion during interval training on changes to muscle buffer capacity, metabolism, and short-term endurance performance. J Appl Physiol 101: 918–925. 16627675 10.1152/japplphysiol.01534.2005

[9] Thomas C, Bishop D, Moore-Morris T, Mercier J (2007) Effects of high-intensity training on MCT1, MCT4, and NBC expressions in rat skeletal muscles: influence of chronic metabolic alkalosis. Am J Physiol Endocrinol Metab 293: E916–922. 17609257 10.1152/ajpendo.00164.2007

[10] Bentley D, Newell J, Bishop D (2007) Incremental exercise test design and analysis: implications for performance diagnostics in endurance athletes. Sports Medicine 37: 575–586. 17595153 10.2165/00007256-200737070-00002

[11] Racinais S, Perrey S, Denis R, Bishop D (2010) Maximal power, but not fatigability, is greater during repeated sprints performed in the afternoon. Chronobiol Int 27: 855–864. 10.3109/07420521003668412 20560715

[12] Hollidge-Horvat MG, Parolin ML, Wong D, Jones NL, Heigenhauser GJ (1999) Effect of induced metabolic acidosis on human skeletal muscle metabolism during exercise. Am J Physiol 277: E647–658. 10516124 10.1152/ajpendo.1999.277.4.E647

[13] Pilegaard H, Ordway GA, Saltin B, Neufer PD (2000) Transcriptional regulation of gene expression in human skeletal muscle during recovery from exercise. American Journal of Physiology Endocrinology and Metabolism 279: E806–814. 11001762 10.1152/ajpendo.2000.279.4.E806

[14] Lundby C, Nordsborg N, Kusuhara K, Kristensen KM, Neufer PD, Pilegaard H (2005) Gene expression in human skeletal muscle: alternative normalization method and effect of repeated biopsies. European Journal of Applied Physiology and Occupational Physiology 95: 351–360. 16151837 10.1007/s00421-005-0022-7

[15] Bailey JL, England BK, Long RC Jr, Weissman J, Mitch WE (1995) Experimental acidemia and muscle cell pH in chronic acidosis and renal failure. American Journal of Physiology 269: C706–712. 7573401 10.1152/ajpcell.1995.269.3.C706

[16] Bishop D, Edge J, Mendez-Villanueva A, Thomas C, Schneiker K (2009) High-intensity exercise decreases muscle buffer capacity via a decrease in protein buffering in human skeletal muscle. Pflugers Arch 458: 929–936. 10.1007/s00424-009-0673-z 19415322

[17] Thomas C, Bishop DJ, Lambert K, Mercier J, Brooks GA (2012) Effects of acute and chronic exercise on sarcolemmal MCT1 and MCT4 contents in human skeletal muscles: current status. Am J Physiol Regul Integr Comp Physiol 302: R1–14. 10.1152/ajpregu.00250.2011 22012699

[18] Nowik M, Lecca MR, Velic A, Rehrauer H, Brändli AW, Wagner CA (2008) Genome-wide gene expression profiling reveals renal genes regulated during metabolic acidosis. Physiological Genomics 32: 322–334. 18056784 10.1152/physiolgenomics.00160.2007

[19] Lecker SH, Jagoe RT, Gilbert A, Gomes M, Baracos V, Bailey J, et al (2004) Multiple types of skeletal muscle atrophy involve a common program of changes in gene expression. FASEB Journal 18: 39–51. 14718385 10.1096/fj.03-0610com

[20] Reaich D, Channon SM, Scrimgeour CM, Goodship TH (1992) Ammonium chloride-induced acidosis increases protein breakdown and amino acid oxidation in humans. Am J Physiol Endocrinol Metab 263: E735–739. 10.1152/ajpendo.1992.263.4.E735 1415693

[21] Mutsvangwa T, Gilmore J, Squires JE, Lindinger MI, McBride BW (2004) Chronic metabolic acidosis increases mRNA levels for components of the ubiquitin-mediated proteolytic pathway in skeletal muscle of dairy cows. Journal of Nutrition 134: 558–561. 14988446 10.1093/jn/134.3.558

[22] Sandri M, Sandri C, Gilbert A, Skurk C, Calabria E, Picard A, et al (2004) Foxo Transcription Factors Induce the Atrophy-Related Ubiquitin Ligase Atrogin-1 and Cause Skeletal Muscle Atrophy. Cell 117: 399–412. 15109499 10.1016/s0092-8674(04)00400-3 PMC3619734

[23] Bevington A, Brown J, Butler H, Govindji S, K MK, Sheridan K, et al (2002) Impaired system A amino acid transport mimics the catabolic effects of acid in L6 cells. European Journal of Clinical Investigation 32: 590–602. 12190959 10.1046/j.1365-2362.2002.01038.x

[24] Ishii S, Kihara Y, Shimizu T (2005) Identification of T cell death-associated gene 8 (TDAG8) as a novel acid sensing G-protein-coupled receptor. Journal of Biological Chemistry 280: 9083–9087. 15618224 10.1074/jbc.M407832200

[25] Stathopoulou K, Gaitanaki C, Beis I (2006) Extracellular pH changes activate the p38-MAPK signalling pathway in the amphibian heart. Journal of Experimental Biology 209: 1344–1354. 16547305 10.1242/jeb.02134

[26] Fan M, Rhee J, St-Pierre J, Handschin C, Puigserver P, Lin J, et al (2004) Suppression of mitochondrial respiration through recruitment of p160 myb binding protein to PGC-1alpha: modulation by p38 MAPK. Genes & Development 18: 278–289. 14744933 10.1101/gad.1152204 PMC338281

[27] Hayashi T, Shigetomi T, Ueda M, Kaneda T, Matsumoto T, Tokuno H, et al (1992) Effects of ammonium chloride on membrane currents of acinar cells dispersed from the rat parotid gland. Pflugers Archiv European Journal of Physiology 420: 297–301. 1598185 10.1007/BF00374462

[28] Rama Rao KV, Jayakumar AR, Norenberg MD (2005) Role of oxidative stress in the ammonia-induced mitochondrial permeability transition in cultured astrocytes. Neurochemistry International 47: 31–38. 15908047 10.1016/j.neuint.2005.04.004

[29] Hashimoto T, Hussien R, Oommen S, Gohil K, Brooks GA (2007) Lactate sensitive transcription factor network in L6 cells: activation of MCT1 and mitochondrial biogenesis. FASEB J 21: 2602–2612. 17395833 10.1096/fj.07-8174com

[30] Egan B, Carson BP, Garcia-Roves PM, Chibalin AV, Sarsfield FM, Barron N, et al (2010) Exercise intensity-dependent regulation of peroxisome proliferator-activated receptor γ coactivator-1α mRNA abundance is associated with differential activation of upstream signalling kinases in human skeletal muscle. The Journal of Physiology 588: 1779–1790. 10.1113/jphysiol.2010.188011 20308248 PMC2887994

[31] Psilander N, Wang L, Westergren J, Tonkonogi M, Sahlin K (2010) Mitochondrial gene expression in elite cyclists: effects of high-intensity interval exercise. EUROPEAN JOURNAL OF APPLIED PHYSIOLOGY 110: 597–606. 10.1007/s00421-010-1544-1 20571821

[32] Morton JP, Croft L, Bartlett JD, Maclaren DP, Reilly T, Evans L, et al (2009) Reduced carbohydrate availability does not modulate training-induced heat shock protein adaptations but does upregulate oxidative enzyme activity in human skeletal muscle. Journal of Applied Physiology 106: 1513–1521. 10.1152/japplphysiol.00003.2009 19265068

[33] Jones NL, Sutton JR, Taylor R, Toews CJ (1977) Effect of pH on cardiorespiratory and metabolic responses to exercise. Journal of Applied Physiology 43: 959–964. 24031 10.1152/jappl.1977.43.6.959

[34] Kowalchuk JM, Heigenhauser GJ, Jones NL (1984) Effect of pH on metabolic and cardiorespiratory responses during progressive exercise. Journal of Applied Physiology 57: 1558–1563. 6520052 10.1152/jappl.1984.57.5.1558

[35] Hardie DG, Sakamoto K (2006) AMPK: A Key Sensor of Fuel and Energy Status in Skeletal Muscle. Physiology 21: 48–60. 16443822 10.1152/physiol.00044.2005

[36] Zong H, Ren JM, Young LH, Pypaert M, Mu J, Birnbaum MJ, et al (2002) AMP Kinase Is Required for Mitochondrial Biogenesis in Skeletal Muscle in Response to Chronic Energy Deprivation. Proceedings of the National Academy of Sciences of the United States of America 99: 15983–15987. 12444247 10.1073/pnas.252625599 PMC138551

[37] Ludwig MG, Vanek M, Guerini D, Gasser JA, Jones CE, Junker U, et al (2003) Proton-sensing G-protein-coupled receptors. Nature 425: 93–98. 12955148 10.1038/nature01905

[38] Shi Q, Le X, Wang B, Abbruzzese JL, Xiong Q, He Y, et al (2001) Regulation of vascular endothelial growth factor expression by acidosis in human cancer cells. Oncogene 20: 3751–3756. 11439338 10.1038/sj.onc.1204500

[39] Caso G, Garlick BA, Casella GA, Sasvary D, Garlick PJ (2004) Acute metabolic acidosis inhibits muscle protein synthesis in rats. American Journal of Physiology 287: E90–E96. 14982751 10.1152/ajpendo.00387.2003

[40] Regensteiner JG, Bauer TA, Reusch JEB, Brandenburg SL, Sippel JM, Vogelsong AM, et al (1998) Abnormal oxygen uptake kinetic responses in women with type II diabetes mellitus. J Appl Physiol 85: 310–317. 9655791 10.1152/jappl.1998.85.1.310

