# Supplementary material for: Ammonium Chloride Ingestion Attenuates Exercise-Induced mRNA Levels in Human Muscle
Source: PLoS One. 2015 Dec 10;10(12):e0141317. doi: 10.1371/journal.pone.0141317 (PMC4686080; doi:10.1371/journal.pone.0141317)
Supplement: S1 Dataset — (PDF) [file pone.0141317.s001.pdf]

S1 Dataset. Raw data for individual participants.

Raw Data - muscle pH

	**Trial PLAC**			**Trial ACID**		
Subject	Pre	Post	"+2h"	Pre	Post	"+2h"
1	7.07	6.91	6.98	7.07	6.95	7.09
2	7.16	6.97	7.11	7.16	7.02	7.16
3	7.10	6.92	7.09	7.08	6.99	7.19
4	7.12	6.92	7.10	7.12	6.99	7.16
5	7.10	6.76	7.00	7.07	6.82	7.07
6	7.06	6.69	7.06	7.10	6.75	7.10
7	7.06	6.90	7.06	7.06	6.95	7.08
8	7.11	6.84	7.02	7.13	6.96	7.12
Mean	7.10	6.86	7.05	7.10	6.93	7.12
SD	0.04	0.11	0.05	0.04	0.09	0.04

Raw Data - PGC1a mRNA (a.u.)

	**Trial PLAC**			**Trial ACID**		
Subject	Pre	Post	"+2h"	Pre	Post	"+2h"
1	0.000686	0.010217	0.121727	0.000470	0.014118	0.042041
2	0.001147	0.011167	0.042451	0.002342	0.001615	0.111733
3	0.002684	0.007635	0.066192	0.020487	0.000591	0.004734
4	0.004169	0.009878	0.103196	0.005675	0.008082	0.000410
5	0.000992	0.002475	0.051687	0.071904	0.002811	0.019440
6	0.014628	0.012868	0.117469	0.000962	0.011746	0.076287
7	0.000376	0.007450	0.018349	0.002751	0.006761	0.014600
8	0.000304	0.016539	0.089411	0.008062	0.009108	0.074505
Mean	0.003	0.010	0.076	0.014	0.007	0.043
SD	0.005	0.004	0.038	0.024	0.005	0.040
SE	0.002	0.001	0.013	0.009	0.002	0.014

Raw Data - CS mRNA (a.u.)

	**Trial PLAC**			**Trial ACID**		
Subject	Pre	Post	2h	Pre	Post	2h
1	0.001242	0.044823	0.040652	0.001222	0.039034	0.029560
2	0.009946	0.036039	0.030875	0.021732	0.010941	0.041022
3	0.018810	0.024368	0.031789	0.047121	0.003104	0.007154
4	0.026038	0.026111	0.031894	0.025810	0.022181	0.001040
5	0.003315	0.011306	0.023993	0.026866	0.013926	0.035472
6	0.054277	0.036150	0.033996	0.012188	0.035094	0.034681
7	0.006782	0.047399	0.020686	0.021313	0.038105	0.021957
Mean	0.017	0.032	0.031	0.022	0.023	0.024
SD	0.019	0.013	0.007	0.014	0.014	0.015

SE	0.007	0.004	0.002	0.005	0.005	0.005

Raw Data – CYTC mRNA (a.u)

	Trial PLAC			Trial ACID		
Subject	Pre	Post	2h	Pre	Post	2h
1	0.003808	0.038483	0.034862	0.003219	0.036607	0.026448
2	0.017436	0.038353	0.028776	0.037944	0.026700	0.046373
3	0.024389	0.025064	0.026228	0.045870	0.006864	0.010759
4	0.031234	0.026391	0.029785	0.031982	0.026209	0.003476
5	0.005702	0.015785	0.021607	0.033215	0.017522	0.025325
6	0.056788	0.037012	0.034911	0.019569	0.034413	0.027483
7	0.006080	0.035772	0.021041	0.020800	0.034886	0.021061
8	0.001644	0.045758	0.033383	0.025273	0.017112	0.021362
Mean	0.018	0.033	0.029	0.027	0.025	0.023
SD	0.019	0.010	0.006	0.013	0.011	0.013
SE	0.007	0.003	0.002	0.005	0.004	0.004

Raw Data – PGC1b mRNA (a.u)

	Trial PLAC			Trial ACID		
Subject	Pre	Post	"+2h"	Pre	Post	"+2h"
1	0.012654	0.024733	0.024484	0.033882	0.020999	0.022402
2	0.027458	0.021146	0.042109	0.027168	0.027658	0.038643
3	0.015103	0.014993	0.027531	0.014707	0.007498	0.019001
4	0.020931	0.015098	0.028347	0.023817	0.010056	0.021150
5	0.019906	0.027933	0.034380	0.025982	0.018563	0.027758
6	0.030449	0.020268	0.028855	0.017437	0.020330	0.032866
7	0.013309	0.025449	0.024935	0.019120	0.018626	0.034302
8	0.008379	0.029477	0.039758	0.019042	0.021727	0.028420
Mean	0.019	0.022	0.031	0.023	0.018	0.028
SD	0.008	0.005	0.007	0.006	0.006	0.007
SE	0.003	0.002	0.002	0.002	0.002	0.002

Raw Data – HKII mRNA (a.u)

	Trial PLAC			Trial ACID		
Subject	Pre	Post	2h	Pre	Post	2h
1	0.008543	0.048781	0.072321	0.013981	0.067447	0.064715
2	0.018254	0.095888	0.096629	0.053795	0.026326	0.145424
3	0.016663	0.031891	0.052055	0.067834	0.003450	0.011216
4	0.017012	0.029783	0.044497	0.021785	0.027972	0.002228
5	0.005990	0.037881	0.047495	0.047757	0.013463	0.039897
6	0.092377	0.072499	0.071135	0.028556	0.082249	0.084125
7	0.036637	0.027895	0.061026	0.073561	0.088247	0.031644
8	0.063600	0.076469	0.090215	0.051887	0.054701	0.057866
Mean	0.032	0.053	0.067	0.045	0.045	0.055
SD	0.031	0.026	0.019	0.022	0.032	0.046
SE	0.011	0.009	0.007	0.008	0.011	0.016

Raw Data – GLUT4 mRNA (a.u.)

	Trial PLAC			Trial ACID		
Subject	Pre	Post	2h	Pre	Post	2h
1	0.0031	0.0287	0.0266	0.001862	0.024778	0.023716
2	0.0110	0.0267	0.0222	0.022641	0.013559	0.030165
3	0.0163	0.0183	0.0225	0.022426	0.004204	0.011742
4	0.0218	0.0194	0.0244	0.013461	0.007466	0.033718
5	0.0075	0.0119	0.0167	0.025411	0.028859	0.00224
6	0.0560	0.0343	0.0318	0.013069	0.034052	0.037158
7	0.0070	0.0397	0.0222	0.022099	0.027507	0.027341
8	0.0089	0.0370	0.0381	0.028188	0.02985	0.032982
Mean	0.016	0.027	0.026	0.019	0.021	0.025
SD	0.017	0.010	0.007	0.009	0.011	0.012
SE	0.006	0.003	0.002	0.003	0.004	0.004

Raw Data – PDK4 mRNA (a.u.)

	Trial PLAC			Trial ACID		
Subject	Pre	Post	"+2h"	Pre	Post	"+2h"
1	0.000108	0.017929	0.035726	0.000550	0.083653	0.108527
2	0.008412	0.100240	0.088361	0.016623	0.007085	0.101995
3	0.008142	0.035648	0.013810	0.001772	0.054119	0.016732
4	0.004984	0.009640	0.010079	0.003242	0.004928	0.000100
5	0.002058	0.023321	0.165031	0.017655	0.019611	0.115158
6	0.052139	0.097969	0.082912	0.005024	0.053469	0.083735
7	0.000691	0.025902	0.041444	0.001267	0.026461	0.031505
8	0.000100	0.024016	0.053491	0.020624	0.038266	0.103851
Mean	0.010	0.042	0.061	0.008	0.036	0.070
SD	0.018	0.036	0.051	0.008	0.027	0.046
SE	0.006	0.013	0.018	0.003	0.010	0.016

Raw Data – FOXO1 mRNA (a.u.)

	Trial PLAC			Trial ACID		
Subject	Pre	Post	"+2h"	Pre	Post	"+2h"
1	0.003088	0.044481	0.051481	0.005145	0.035460	0.048344
2	0.024267	0.044727	0.061151	0.018616	0.017704	0.070546
3	0.012849	0.028754	0.025870	0.039271	0.004291	0.040115
4	0.019952	0.024747	0.042508	0.018970	0.024080	0.003289
5	0.008072	0.034754	0.074711	0.048574	0.029808	0.091463
6	0.044469	0.036236	0.089353	0.019658	0.034689	0.058909
7	0.007153	0.032038	0.040636	0.030236	0.035464	0.035927
8	0.001124	0.039268	0.056723	0.032305	0.056744	0.059353
Mean	0.015	0.036	0.055	0.027	0.030	0.051
SD	0.014	0.007	0.020	0.014	0.015	0.026

SE	0.005	0.003	0.007		0.005	0.005	0.009
